# Inequality and mental healthcare utilisation among first-year university students in South Africa

**DOI:** 10.1186/s13033-020-0339-y

**Published:** 2020-01-25

**Authors:** Jason Bantjes, Wylene Saal, Christine Lochner, Janine Roos, Randy P. Auerbach, Philippe Mortier, Ronny Bruffaerts, Ronald C. Kessler, Dan J. Stein

**Affiliations:** 1 Department of Psychology, Stellenbosch University, Private Bag X1, Matieland, 7602 South Africa; 2 MRC Unit on Risk and Resilience in Mental Disorders, Department of Psychiatry, Stellenbosch University, Stellenbosch, South Africa; 3 Division of Child and Adolescent Psychiatry, Columbia University, New York, USA; 4 Health Services Research Group, IMIM (Hospital del Mar Medical Research Institute), Barcelona, Spain; 5 CIBER Epidemiología y Salud Pública (CIBERESP), Madrid, Spain; 6 Research Group Psychiatry, Department of Neurosciences, KU Leuven University, Louvain, Belgium; 7 Universitair Psychiatrisch Centrum - Katholieke Universiteit Leuven (UPC-KUL), Campus Gasthuisberg, Louvain, Belgium; 8 Department of Healthcare Policy, Harvard Medical School, Boston, MA USA; 9 MRC Unit on Risk and Resilience in Mental Disorders, Department of Psychiatry and Mental Health, University of Cape Town, Cape Town, South Africa

**Keywords:** Mental healthcare utilisation, Treatment, Common mental disorders, University students, South Africa

## Abstract

**Background:**

Addressing inequalities in mental healthcare utilisation among university students is important for socio-political transformation, particularly in countries with a history of educational exclusion.

**Methods:**

As part of the WHO World Mental Health International College Student Initiative, we investigated inequalities in mental healthcare utilisation among first-year students at two historically “White” universities in South Africa. Data were collected via a web-based survey from first-year university students (n = 1402) to assess 12-month mental healthcare utilisation, common mental disorders, and suicidality. Multivariate logistic regression models were used to estimate associations between sociodemographic variables and mental healthcare utilisation, controlling for common mental disorders and suicidality.

**Results:**

A total of 18.1% of students utilised mental healthcare in the past 12 months, with only 28.9% of students with mental disorders receiving treatment (ranging from 28.1% for ADHD to 64.3% for bipolar spectrum disorder). Of those receiving treatment, 52.0% used psychotropic medication, 47.3% received psychotherapy, and 5.4% consulted a traditional healer. Treatment rates for suicidal ideation, plan and attempt were 25.4%, 41.6% and 52.9%, respectively. In multivariate regression models that control for the main effects of mental health variables and all possible joint effects of sociodemographic variables, the likelihood of treatment was lower among males (aOR = 0.57) and Black students (aOR = 0.52). An interaction was observed between sexual orientation and first generation status; among second-generation students, the odds of treatment were higher for students reporting an atypical sexual orientation (aOR = 1.55), while among students with atypical sexual orientations, the likelihood of mental healthcare utilisation was lower for first-generation students (aOR = 0.29). Odds of treatment were significantly elevated among students with major depressive disorder (aOR = 1.88), generalised anxiety disorder (aOR = 2.34), bipolar spectrum disorder (aOR = 4.07), drug use disorder (aOR = 3.45), suicidal ideation (without plan or attempt) (aOR = 2.00), suicide plan (without attempt) (aOR = 3.64) and suicide attempt (aOR = 4.57). Likelihood of treatment increased with level of suicidality, but not number of mental disorders.

**Conclusion:**

We found very low mental healthcare treatment utilisation among first-year university students in South Africa, with enduring disparities among historically marginalised groups. Campus-based **i**nterventions are needed to promote mental healthcare utilisation by first-year students in South Africa, especially among male and Black students and first-generation students with atypical sexual orientations.

## Inequality and mental healthcare utilisation among first-year university students in South Africa

Mental health problems are common among university students globally, with the prevalence of 12-month common mental disorders estimated to be above 30% in many universities [1]. Student mental health problems are associated with a range of adverse outcomes, including severe role impairment [2], academic failure [3], and suicidal behaviour [4]. Early intervention and effective treatment lead to improved outcomes and reduce the morbidity and mortality associated with mental disorders. Yet, the mental health treatment gap among university students is marked; data from 21 countries collected as part of the World Health Organization (WHO) World Mental Health Surveys indicated that an average of only 6.4% of students with 12-month mental disorders received treatment in the preceding 12 months [5]. Patterns of mental health service utilisation among students are shaped by the accessibility of appropriate services and students’ perception of their need for services. Sociodemographic and economic factors also influence mental healthcare utilisation, with considerable racial and gender inequalities in access to mental healthcare among university students [6–9]. To plan effective and efficient student mental health systems, it is essential to understand patterns of mental healthcare utilisation and identify groups of students who may be excluded from receiving mental healthcare. Addressing mental healthcare disparities among students is important from a human rights and transformation perspective, particularly in countries like South Africa (SA) where the political history of the country has created endemic and enduring inequality in access to healthcare, education, and economic opportunities. Inequality in mental healthcare utilisation among university students threatens transformation and economic development by perpetuating social inequality and maintaining groups of students in marginalized and precarious positions. In preparation for developing a series of interventions to address these problems, we carried out a survey to estimate prevalence of common mental disorders and correlates of mental healthcare utilisation among first-year students at two historically “White” universities in SA. This work was carried out as part of the WHO World Mental Health International College Student Initiative (WMH-ICS) [10]. We were particularly interested in potential inequalities in service utilisation among groups of students that have been historically marginalized from higher education in SA, including those who identify as Black, female, disabled, and first-generation students.

### Student mental healthcare utilisation

Studies consistently report low treatment rates among university students with mental health problems [9]. Zivin et al. [11] found that fewer than half of US students (n = 763) with a mental disorder received treatment, while a survey of students in Lebanon (n = 543) found that formal health-care-seeking behaviour was almost non-existent for psychological disorders (3.3%), relational and social issues (1.8%), or substance use problems (5.1%) [4]. Students’ utilisation of mental health service varies across different mental disorders [9], with treatment rates being particularly low for depressive disorders [12, 13]. The low rates of treatment seeking observed among university students are due, at least in part, to difficulties accessing services, but data also suggest that undergraduate students are often strongly disinclined to seek formal treatment from a mental health professional, preferring instead to access psycho-social support from friends, family, or via self-help [14].

### Sociodemographic determinants of mental healthcare utilisation

Patterns of mental healthcare utilisation in the general population are a function of sociodemographic factors, such as ethnicity, gender, sexual orientation and socio-economic status. The patterns of mental healthcare utilisation observed in the general population are also broadly reflected in student populations, although some data suggests that there may be particular context-sensitive factors on university campuses, such as social stigma or perceptions about confidentiality, that prohibit some students from accessing care [6–9].

Marked ethnic and racial inequalities in mental health utilisation are well documented. Studies in the general population of the US suggest that Black Americans seek mental health services at much lower rates than White Americans; the reasons for this difference include socio-cultural barriers to care (such as stigma, lack of culturally relevant treatment models, and attitudes toward mental health services) as well as systemic structural barriers (such as systematic oppression, institutionalized racism, and structural disparities) [15]. Similar patterns of ethnic and racial inequalities in mental healthcare utilisation are observed on university campuses [16–18]. A large study of 43,375 undergraduate and graduate students from 60 institutions in the USA found significantly lower odds of mental health treatment utilisation among students of colour compared to their White peers, even when controlling for other variables in regression models [17]. Similarly, a survey of 2785 students attending a large, public university in the USA found significantly lower mental health service utilisation rates among ethnic minorities [12], and a study of students at the University of Hawaii (n = 589) found that among students with high levels of depressive symptoms, European Americans were 3.7 times as likely as other students to receive mental health treatment in the previous 12 months [13].

Rates of mental healthcare utilisation in the general population are typically higher among women than men, a pattern also found consistently among college students [6, 7]. Comparatively less attention has been paid to patterns of mental health service utilisation among gay, lesbian or bisexual students. Although there is some evidence that students identifying as gay and bisexual are more inclined to utilise mental health services compared to their heterosexual peers [8], this might reflect greater need for services rather than a higher predisposition to seek help, and the failure to adjust for differential need might even mask a lower predisposition to seeking help among this subgroup of students.

Although there is a growing body of literature on the factors that shape student mental healthcare utilisation [5–9, 14, 17], the research in this area has at least three important limitations. First, much of the literature is confined to high income western countries, with a dearth of studies from Africa. Second, interactions between sociodemographic factors associated with treatment seeking are not well documented. Third, the relationship between treatment seeking and suicidality has not been thoroughly explored.

## Methods

The aims of this study were to: (1) determine the prevalence of 12-month mental health care utilisation among first-year university students in SA; (2) establish the range of mental health care services accessed by SA university students; (3) investigate interactions between sociodemographic variables as determinants of mental healthcare utilisation; and (4) document sociodemographic disparities in mental healthcare utilisation among students with mental health problems and suicidality.

### Procedure

Data for this cross-sectional study were collected via an online self-report survey of first-year students at two well-resourced historically “White” universities in SA. Both of these institutions have free campus-based student mental health services. We invited all first year students via email to participate in the study (N = 14 575), of which 53.7% (n = 7827) were women and 43.1% Black (n = 6282). A total of 1407 students completed the survey (yielding a participation rate of 9.7%), although data for 5 participants could not be used because they chose not to provide key sociodemographic data required for the analysis in this study.

### Data collection

The following data were collected using the survey instrument developed for use in the WHO World Mental Health International College Student Initiative [2]:

1. *Sociodemographic characteristics* In addition to questions about age, parents’ level of education and health, participants were asked how they self-identify in terms of gender, sexual orientation, and population group. Participants were identified as “first-generation students” (if neither of their parents had completed tertiary education) or as “second-generation students” (if either of their parents had obtained a university degree). Students were identified as having a disability if they reported any chronic illnesses (e.g., diabetes, asthma, chronic pain disorder, or migraines), or any severe physical impairment (e.g., vision, hearing, or movement impairment). Sexual orientation was dichotomised into “typical” (i.e. no same sex attraction) or “atypical” (i.e. lesbian, gay, bisexual, asexual or unsure). Population group was coded as “Black” or “White”; the term “Black” was used to denote all students who identified as Coloured (an official term used for census data and population classification in SA), Black-African or Indian. This broad definition of Black was used to identify all students who have historically been excluded from universities in SA; the use of these terms is not intended to reify sociocultural constructs about ethnicity, but rather to investigate enduring inequalities among historically marginalised population groups.
2. *Mental healthcare utilisation* Participants were asked if they had accessed treatment in the past 12 months for an emotional or substance use problem. They were also asked if their treatment had entailed psychological counselling, and/or the use of medication, and/or consulting a traditional healer.
3. *Common mental disorders* We assessed whether participants scored positive in the past 12 months for six common mental disorders: major depressive disorder (MDD), generalized anxiety disorder (GAD), bipolar spectrum disorder, alcohol use disorder (AUD), drug use disorder (DUD), and ADHD [19]. Survey instruments used to assess these disorders were drawn from the CIDI, EPI-Q Screening Survey [20], Alcohol Use Disorders Identification Test [21], and The World Health Organization Adult ADHD Self-Report Scale [22]. Caseness for mental disorders was determined using the procedure validated in the Army Study to Assess Risk and Resilience in Service Members (Army STARRS) [23], and repeated in the WHO World Mental Health Surveys and WMH-ICS Initiative [10].
4. *Suicidal thoughts and behavior* Items from the Columbia Suicidal Severity Rating Scale [24] were used to assess 12-month prevalence of: (1) suicidal ideation (“*Did you wish you were dead or would go to sleep and never wake up?”* or *“Did you have thoughts of killing yourself?*”); (2) suicide plan (“*Did you think about how you might kill yourself [e.g., taking pills, shooting yourself] or work out a plan of how to kill yourself?*”); and (3) suicide attempt (“*Have you made a suicide attempt [i.e., purposefully hurt yourself with at least some intent to die]?*”).

### Data analysis

Data were checked, cleaned and imported into SPSS 25 for analysis. To adjust for non-response bias, data were weighted by gender and population group using a post-stratification weighting technique [25]. Descriptive statistics were used to document mental health treatment rates among participants who reported a common mental disorder or suicidal thoughts and behaviours. Bivariate and multivariate regression analysis was used to identify associations between sociodemographic factors and mental healthcare utilisation, exploring all main effects and all possible two-by-two interaction effects. In the final step of the analysis we estimated multivariate regression models to investigate sociodemographic correlates of mental healthcare utilisation, controlling for common mental disorders and suicidality. The results of all regression analyses are reported as adjusted odds ratios (aOR) with associated 95% Confidence Intervals (95% CI). The significance level was set at alpha = 0.05 for all statistical analysis.

### Ethics

We obtained ethical approval from the institutional review boards of both universities. Participation in the study was entirely voluntary and informed consent was obtained from all participants prior to data collection. Participants received information about counselling and crises services. All data were anonymised and securely stored in password protected cloud-based servers.

## Results

### Sample characteristics

The sample was constituted predominantly by students under 21 years of age (92.3%), who identified as female (55.2%), White (58.6%), heterosexual (77.8%), able-bodied (81.6%), and second-generation students (80.3%). A total of 42.7% met criteria in the preceding 12 months for at least one of the common mental disorders assessed, and 18.1% reported utilising mental healthcare in the past 12 months. Among those who accessed mental healthcare, 52.0% made use of psychotropic medication, 47.3% received psychotherapy, and 5.4% sought treatment from a traditional healer for their mental health problems. Elsewhere we have reported on the prevalence and sociodemographic correlates of common mental disorders in this sample [26], and on the epidemiology of non-fatal suicidal behaviour [27]. Below we present an analysis of factors associated with utilisation of mental healthcare in this sample.

### Mental healthcare utilisation among students with mental health problems

Only 28.9% of students with a mental disorder received treatment in the preceding 12 months (see Table 1). Treatment rates ranged from a low of 28.1% for ADHD to a high of 64.3% for bipolar spectrum disorder. Treatment rates increased with the number of disorders; the proportion of students with exactly one, two and three or more disorders receiving treatment was 22.7%, 30.2% and 47.9%, respectively. Only 35.0% of students who reported suicidal thoughts or behaviours in the past 12 months utilised mental healthcare; treatment rates among students who reported suicidal ideation (without plan or attempt), suicide plan (without attempt) and suicide attempt were 25.4%, 41.6% and 52.9%, respectively.

**Table 1:** Twelve-month mental healthcare utilisation associated with mental health problems among first-year university students in South Africa (n = 1402)

	Predictor distribution in sample (95% CI)	Proportion utilising mental healthcare % (95% CI)	Multivariate analysis of associations with mental healthcare utilisation aOR (95% CI)
Type of mental disorder			
Major depressive disorder	13.6% (11.9–15.5)	36.1% (29.3–43.4)	1.98* (1.36–2.88)
Generalized anxiety disorder	20.8% (18.7–23.0)	36.1% (30.6–41.9)	2.50* (1.78–3.52)
Bipolar spectrum disorder	1.0% (0.6–1.7)	64.3% (35.2–87.3)	4.97* (1.45–17.09)
Alcohol use disorder	5.6% (4.45–6.94)	21.8% (13.2–32.6)	0.85 (0.45–1.58)
Drug use disorder	3.1% (2.3–4.15)	55.8% (39.9–70.9)	4.55* (2.30–8.98)
Attention deficit hyperactivity disorder	25.9% (23.6–28.3)	28.1% (23.5–33.0)	1.32 (0.95–1.84)
			R^2^ = 0.132
			X^2^ (6) = 118.39
			p = 0.00*
Number of disorders			
Exactly one mental disorder	23.6% (21.4–25.9)	22.7% (18.3–27.6)	2.61* (1.85-3.69)
Exactly two mental disorders	12.3% (10.6–14.1)	30.2% (23.4–37.7)	3.85* (2.59-5.74)
Three or more mental disorders	6.9% (5.6–8.4)	47.9% (37.6–58.3)	8.15* (5.14-12.93)
			R^2^ = 0.116
			X^2^ (3) = 103.30
			p = 0.00*
Suicidal thoughts and behaviors			
Suicidal ideation without plan and without attempt	14.0% (12.2–15.9)	25.4% (19.5–32.1)	2.85* (1.95–4.16)
Suicidal plan without attempt	14.1% (12.3–16.0)	41.6% (34.6–48.8)	5.96* (4.21–8.44)
Suicide attempt	2.4% (1.7–3.3)	52.9% (35.1–70.2)	9.16* (4.55–18.43)
			R^2^ = 0.141
			X^2^ (3) = 126.72
			p = 0.00*

*aOR* adjusted odds ratio, *CI* confidence interval

*p < 0.05

In multivariate regression analysis of associations between 12-month mental healthcare utilisation and mental disorders, receiving treatment was significantly associated with MDD (aOR = 1.98, 95% CI = 1.36–2.88), GAD (aOR = 2.50, 95% CI = 1.78–3.52), bipolar spectrum disorder (aOR = 4.97, 95% CI = 1.45–17.09) and DUD (aOR = 4.55, 95% CI = 2.30–8.98), but not with any other disorders assessed (Table 1). In addition, the multivariate regression analysis of associations between 12-month mental healthcare utilisation and the number of disorders, the odds of receiving treatment were 2.61 (95% CI = 1.85–3.69) for exactly one mental disorder, 3.85 (95% CI = 2.59–5.74) for two disorders, and 8.15 (95% CI = 5.14–12.93) for three or more disorders (Table 1). In multivariate regression analysis of associations between 12-month mental healthcare utilisation and suicidal thoughts and behaviours, the odds of receiving treatment among students who reported suicidal ideation (without plan or attempt), suicide plan (without attempt) and suicide attempt were 2.85 (95% CI = 1.95–4.16), 5.96 (95% CI = 4.21–8.44), and 9.16 (95% CI = 4.55–18.43), respectively (Table 1).

The results of multivariate regression analysis of associations between 12-month mental healthcare utilisation and all mental health variables (i.e. the six common mental disorders assessed, the number of disorders, and all dimensions of suicidality) are presented in Table 2. Receiving treatment was significantly associated with MDD (aOR = 1.89; 95% CI = 1.12–3.20), GAD (aOR = 2.68; 95% CI = 1.65–4.37), DUD (aOR = 3.93, 95% CI = 1.78–8.66), suicidal ideation (without plan or attempt) (aOR = 2.05, 95% CI = 1.37–3.08), suicide plan (without attempt) (aOR = 3.69, 95% CI = 2.51–5.43) and suicide attempt (aOR = 4.45, 95% CI = 2.08–9.59), controlling for all other mental health variables in the model.

**Table 2:** Multivariate regression analysis of associations between 12-month mental healthcare utilisation and common mental disorders

	Predictor distribution % (95% CI)	aOR (95% CI)
Major depressive disorder	13.6% (11.9–15.5)	1.89* (1.12–3.20)
Generalized anxiety disorder	20.8% (18.7–23.0)	2.68* (1.65–4.37)
Bipolar spectrum disorder	1.0% (0.6–1.7)	3.64 (0.98–13.47)
Alcohol use disorder	5.6% (4.45–6.94)	1.03 (0.52–2.02)
Drug use disorder	3.1% (2.3–4.15)	3.93* (1.78–8.66)
Attention deficit hyperactivity disorder	25.9% (23.6–28.3)	1.44 (0.93–2.23)
Exactly two disorders	12.3% (10.6–14.1)	0.57 (0.29–1.12)
Three or more disorders	6.9% (5.6–8.4)	0.46 (0.16–1.33)
Suicidal ideation without plan and without attempt	14.0% (12.2–15.9)	2.05* (1.37–3.08)
Suicidal plan without attempt	14.1% (12.3–16.0)	3.69* (2.51–5.43)
Suicide attempt	2.4% (1.7–3.3)	4.45* (2.08–9.59)
		R^2^ = 0.190; X^2^ (11) = 172.93; p = 0.00*
		X^2^ (2) = 2.71^a^; p = 0.26
		X^2^ (6) = 23.08^b^; p = 0.00*
		X^2^ (3) = 49.36^c^; p = 0.00*

The number of disorders. Suicidal thoughts and behaviour among first-year university students in South Africa (n = 1402)

*aOR* adjusted odds ratio, *CI* confidence interval

*p < 0.05

^a^2 degree of freedom test for exactly 2 disorders and 3 or more disorders

^b^6 degree of freedom test for the six common mental disorders in the model

^c^3 degree of freedom test for the three suicidality variables in the model

### Sociodemographic correlates of mental healthcare utilisation

The results of the bivariate and multivariate analysis of sociodemographic factors associated with 12-month mental healthcare utilisation are presented in Table 3. In the multivariate model of main effects, the odds of receiving treatment were significantly higher among students who identified as female (aOR = 2.06, 95% CI = 1.53–2.78), students with atypical sexual orientation (aOR = 1.81, 95% CI = 1.29–2.52), and students who are disabled (aOR = 1.41, 95% CI = 1.01–1.98), but were lower among students who identified as Black (aOR = 0.63, 95% CI = 0.46–0.86) and first-generation students (aOR = 0.52, 95% CI = 0.33–0.80).

**Table 3:** Bivariate and multivariate analysis of sociodemographic correlates of twelve-month mental healthcare utilisation among first-year university students in South Africa (n = 1402)

	Predictor distribution (95% CI)	Bivariate associations with 12-month treatment seeking OR (95% CI)	Multivariate associations with 12-month treatment seeking aOR (95% CI)
Gender (female)	55.2% (52.6–57.8)	2.08* (1.55–2.78)	2.06* (1.53–2.78)
Population group (black)	41.4% (38.8–44.0)	0.61* (0.46–0.81)	0.63* (0.46–0.86)
First generation students	19.7% (17.7–21.9)	0.51* (0.34–0.76)	0.52* (0.33–0.80)
Sexual orientation (atypical sexual orientation)	22.2% (20.1–24.5)	1.39* (1.02–1.90)	1.81* (1.29–2.52)
Disability	18.4% (16.4–20.5)	1.53* (1.10–2.11)	1.41* (1.01–1.98)
			R^2^ = 0.068
			X^2^ (6) = 59.94
			p = 0.00*

*OR* odds ratio, *aOR* adjusted odds ratio, *CI* confidence interval

*p < 0.05

All possible two-by-two interactions of associations between sociodemographic variables and 12-month mental healthcare utilisation were explored (see Additional file 1: Table S1). A significant interaction was observed between sexual orientation and being a first-generation student (see Additional file 1: Table S2). This interaction was explored in multivariate regression models (see Additional file 1: Tables S3, S4), to identify the best fitting model of the joint effects of sociodemographic predictors of treatment (Table 4). The odds of mental healthcare utilisation were significantly higher among students who identified as female (aOR = 2.14, 95% CI = 1.59–2.87), but were significantly lower among students who identified as Black (aOR = 0.62, 95% CI = 0.46–0.85). Among second-generation students, the odds of mental healthcare utilisation were higher for students reporting atypical sexual orientation (compared to those with typical sexual orientations) (aOR = 2.37, 95% CI = 1.65–3.39). Among students with atypical sexual orientations, the likelihood of mental healthcare utilisation was lower for first-generation students (compared to second-generation students) (aOR = 0.21, 95% CI = 0.10–0.44).

**Table 4:** Multivariate analysis of multivariate analysis of sociodemographic correlates of twelve-month mental healthcare utilisation among first-year university students in South Africa (n = 1402)

	Predictor distribution (95% CI)	12-month treatment seeking aOR (95% CI)	12-month treatment seeking aOR (95% CI)
Gender (female)	55.2% (52.6–57.8)	2.14* (1.59–2.87)	2.14* (1.59–2.87)
Population group (black)	41.4% (38.8–44.0)	0.62* (0.46–0.85)	0.62* (0.46–0.85)
Atypical sexual orientation versus typical sexual orientation among *second generation students*	16.4% (14.3–18.7)	2.37* (1.65–3.39)	2.37* (1.65–3.39)
First generation versus second-generation students with *typical sexual orientation*	13.7% (11.7–15.9)	0.95 (0.57–1.59)	0.95 (0.57–1.59)
Atypical sexual orientation versus typical sexual orientation among first-generation students	45.7% (39.7–51.8)	–	0.53 (0.24–1.18)
First generation versus second-generation students with *atypical sexual orientation*	40.6% (35.1–46.3)	0.21* (0.10–0.44)	**–**
		R^2^ = 0.077	R^2^ = 0.077
		X^2^ (5) = 67.85	X^2^ (5) = 67.85
		p = 0.00*	p = 0.00*

*aOR* adjusted odds ratio, *CI* confidence interval

*p < 0.05

In order to investigate if the associations between mental healthcare utilisation and the joint effects of sexual orientation and first-generation status observed in Table 4 simply reflect differences in the need for services within these sub-groups, we calculated the prevalence of mental health problems by sexual orientation and first-generation status (Table 5). We found that the prevalence of mental disorders was consistently higher among second-generation students with atypical sexual orientations (compared to second-generation students with typical sexual orientations), and among second-generation students with atypical sexual orientation (compared to first-generation students with typical sexual orientations). Likewise, first-generation students with typical sexual orientations reported a higher prevalence of all mental health problems (compared to first-generation students with atypical sexual orientation), for all mental health conditions accept bipolar spectrum disorder, ADHD, and suicide plan without attempt.

**Table 5:** Prevalence of common mental disorders and suicidal ideation and behaviour among first-year university students in South Africa by first generation status and sexual orientation (n = 1402)

	First-generation typical sexual orientation (n = 150)% (95% CI)	First-generation atypical sexual orientation (n = 126)% (95% CI)	Second- generation students with typical sexual orientation (n = 942)% (95% CI)	Second-generation students with atypical sexual orientation (n = 185)% (95% CI)
Major depressive disorder	22.2% (15.8–29.7)	12.1% (7.0–19.1)	11.0% (9.1–13.2)	21.5% (15.8–28.1)
Generalized anxiety disorder	24.6% (17.9–32.3)	22.4% (15.5–30.7)	18.2% (15.8–20.8)	29.7% (23.2–36.8)
Bipolar spectrum disorder	0.9% (0.1–4.1)	1.1% (0.1–4.9)	0.6% (0.2–1.3)	2.7% (0.9–6.2)
Alcohol use disorder	6.8% (3.3–12.1)	2.7% (0.6–7.3)	5.4% (4.1–7.0)	7.5% (4.2–12.3)
Substance use disorder	2.4% (0.6–6.3)	1.9% (0.3–6.1)	2.3% (1.4–3.5)	8.2% (4.7–13.1)
Attention Deficit Hyperactivity Disorder	32.0% (24.6–40.1)	34.3% (26.1–43.3)	22.7% (20.1–25.5)	31.4% (24.8–38.6)
Exactly 2 disorders	16.9% (11.3–23.9)	13.9% (8.4–21.2)	11.0% (9.1–13.2)	13.9% (9.3–19.7)
Three or more disorders	9.5% (5.3–15.4)	6.2% (2.7–11.9)	5.1% (3.8–6.7)	14.3% (9.6–20.2)
Suicidal ideation without plan and without attempt	14.3% (9.1–20.9)	10.4% (5.7–17.1)	13.8% (11.7–16.2)	17.3% (12.1–23.5)
Suicidal plan without suicide attempt	11.3% (6.7–17.5)	12.3% (7.1–19.3)	12.5% (10.5–14.8)	25.7% (19.6–32.6)
Suicidal attempt	3.7% (1.3–8.1)	1.5% (0.2–5.5)	1.9% (1.1–3.0)	5.0% (2.3–9.2)

### Sociodemographic and mental health correlates of mental healthcare utilisation

In the final step of the analysis, two regression models were constructed to investigate associations of sociodemographic factors with 12-month mental healthcare utilisation, controlling for mental health variables (Table 6). As seen in model 1, the likelihood of receiving treatment was significantly higher among students who identified as female (aOR = 1.75) and among first-generation (compared to second-generation) students with atypical sexual orientation (aOR = 1.55), but lower among students who identified as Black (aOR = 0.52), when controlling for mental disorder type and number. Model 1 also shows that likelihood of utilising mental healthcare was significantly higher among students with MDD (aOR = 1.88), GAD (aOR = 2.34), bipolar spectrum disorder (aOR = 4.07), DUD (aOR = 3.45), suicidal ideation (without plan or attempt) (aOR = 2.00), suicide plan (without attempt) (aOR = 3.64) and suicide attempt (aOR = 4.57), net of the other variables in the model. As seen in model 2, which evaluated joint effects, likelihood of accessing treatment did not increase with the number of mental disorders (X^2^(3) = 2.81; p = 0.42), but did increase with the level of suicidality (X^2^(3) = 43.99; p = 0.00).

**Table 6:** Multivariate analysis of mental health and sociodemographic variables as predictors of twelve-month treatment seeking (n = 1402)

	Predictor distribution % (95% CI)	Model 1 aOR (95% CI)	Model 2 aOR (95% CI)
Gender (female)	55.2% (52.6–57.8)	1.75* (1.27–2.42)	1.75* (1.27–2.42)
Population group (black)	41.4% (38.8–44.0)	0.52* (0.37–0.73)	0.52* (0.37–0.73)
Atypical sexual orientation versus typical sexual orientation among *second- generation students*	16.4% (14.3–18.7)	1.55* (1.04–2.33)	1.55* (1.03–2.32)
First generation versus second- generation students with *typical sexual orientation*	13.7% (11.7–15.9)	0.85 (0.49–1.47)	0.85 (0.49–1.46)
First generation versus second- generation students with *atypical sexual orientation*	40.6% (35.1–46.3)	0.29* (0.13–0.64)	0.29* (0.13–0.65)
Major depressive disorder	13.6% (11.9–15.5)	1.88* (1.10–3.21)	1.59 (0.78–3.24)
Generalized anxiety disorder	20.8% (18.7–23.0)	2.34* (1.42–3.86)	1.97 (0.99–3.92)
Bipolar spectrum disorder	1.0% (0.6–1.7)	4.07* (1.10–15.09)	3.53 (0.90–13.86)
Alcohol use disorder	5.6% (4.45–6.94)	0.99 (0.50–1.95)	–
Drug use disorder	3.1% (2.3**–**4.15)	3.45* (1.53–7.80)	2.89* (1.11–7.50)
Attention deficit hyperactivity disorder	25.9% (23.6**–**28.3)	1.57 (0.99**–**2.46)	1.30 (0.66**–**2.59)
Exactly 1 disorder	23.6% (21.4**–**25.9)	–	1.26 (0.64**–**2.48)
Exactly two disorders	12.3% (10.6**–**14.1)	0.63 (0.32**–**1.24)	0.90 (0.26**–**3.05)
Three or more disorders	6.9% (5.6**–**8.4)	0.48 (0.16**–**1.42)	0.84 (0.13**–**5.47)
Suicidal ideation without plan and without attempt	14.0% (12.2**–**15.9)	2.00* (1.32–3.03)	2.00* (1.32–3.03)
Suicidal plan without attempt	14.1% (12.3**–**16.0)	3.64* (2.44–5.43)	3.58* (2.39–5.35)
Suicide attempt	2.4% (1.7**–**3.3)	4.57* (2.05–10.20)	4.59* (2.06–10.22)
		R^2^ = 0.235; X^2^ (16) = 217.79; p = 0.00*	R^2^ = 0.236; X^2^ (16) = 218.24; p = 0.00*
		X^2^(2) = 2.00^a;^ p = 0.37	X^2^ (3) = 2.81^d;^ p = 0.42
		X^2^ (6) = 19.10^b^; p = 0.00*	X^2^ (5) = 8.78^e^; p = 0.12
		X^2^ (3) = 44.94^c^; p = 0.00*	X^2^ (3) = 43.99^c;^ p = 0.00*

*aOR* adjusted odds ratio, *CI* confidence interval

*p < 0.05

^a^2 degree of freedom test for exactly 2 disorder and three or more disorders

^b^6 degree of freedom test for the six common mental disorders in the model

^c^3 degree of freedom test for the three suicidality variables in the model

^d^3 degree of freedom test for exactly 1 disorder. Exactly 2 disorders and exactly 3 disorders

^e^5 degree of freedom test for the five common mental disorders in the model

## Discussion

The findings of this study provide the first data on mental healthcare utilisation among undergraduate students in SA, and add to the growing body of literature documenting the mental health treatment gap among university students globally [5, 9, 16, 18]. It is striking that among our sample of first-year students from two well-resourced universities in SA, only 28.9% of students with common mental disorders utilised mental healthcare services in the preceding 12 months, in spite of having access to free student counselling services on campus. The treatment rates observed in our sample are lower than the treatment rates typically reported for students in the USA and Europe [9], but marginally higher than the treatment rate of 25% reported for the general population of SA [28]. This finding draws attention to the need for interventions to increase mental healthcare coverage for university students in SA and further research to understand the reasons for low mental healthcare utilisation among this population.

It is noteworthy that among students who sought treatment for a mental health problem, the majority made use of pharmaceutical interventions (52.0%) and a slightly lower proportion utilised psychological interventions (47.3%). It is unclear from this finding, whether pharmacological interventions are preferred or if their higher use reflects that they are more readily available than psychological interventions, but this is an issue that we are exploring in ongoing analyses of the data. In either case, our data suggests that an opportunity exists to expand the range of psychological interventions offered to these students. The fact that 5.4% of students reported having consulted a traditional healer for mental health problems raises the possibility that non-western and non-biomedical mental health interventions may have purchase among some SA students, a possibility that could be explored as complementary (perhaps culturally more appropriate) approaches to improving mental health treatment rates among SA university students.

The low mental healthcare utilisation rates among students with suicidal thoughts and behaviours has important implications for campus-based suicide prevention in SA. The treatment rate we observed of 35.0% among students who reported suicidal thoughts or behaviours in the past 12-months is congruent with findings from other countries [9]. Given that a history of suicidal thoughts and behaviour are significant risk factors for future suicidal behaviour [29], it would make sense to improve mental healthcare utilisation among students with a recent history of suicidality as an integral component of campus-based suicide prevention programmes. Our data strongly suggest that there is a need for targeted outreach to SA students with a history of suicidal thoughts and behaviours and a need to increase these students’ access to evidence based suicide-prevention interventions.

Crucially, our data draw attention to sociodemographic disparities in mental healthcare utilisation among first-year students in SA. It is noteworthy that the likelihood of utilising mental healthcare was significantly lower for male and Black students, and for first-generation students with atypical sexual orientations (compared to second-generation students with atypical sexual orientations), even when controlling for mental health status. These findings suggest that black, male and first-generation students with atypical sexual orientations, face particular barriers to accessing mental healthcare, and require targeted interventions to improve their utilisation of mental health treatments.

Burkett et al. [15] have proposed the theoretical construct of “obstructed use” to highlight structural and institutional barriers to accessing mental healthcare. While it is certainly important to consider the structural obstacles that SA university students face to accessing mental healthcare, it is also important to remember that even in environments with universal access to free short-term psychotherapy and basic health services, most students with mental health problems do not receive treatment [12]. This reality reminds us that in addition to structural and economic barriers to accessing mental healthcare, there are also individual psychological factors that impede treatment seeking among college students, including factors such as high levels of attachment anxiety and self-stigma [22], attitudinal barriers, perception of need, lack of knowledge about available services, and scepticism about treatment effectiveness [19]. Any efforts to increase utilisation of mental healthcare among SA university students will necessitate investigating and addressing individual-level attitudes, beliefs and knowledge which act as barriers to accessing campus-based mental healthcare. In this context it is interesting to note that in a systematic review of mental health service research in SA, Petersen and Lund noted the need for promoting culturally congruent services as well as mental health literacy to increase help-seeking behaviour, reduce stigma, improve adherence, and eradicate human rights abuses within the delivery of mental healthcare in the country [30]. In the past 10 years a number of scholars have advocated for the implementation of cost-effective culturally-appropriate mental health services and the use of task shifting and stepped care approaches to improving the treatment of common mental disorders in SA [30, 31]. These broad recommendations are also appropriate as strategies to develop campus-based mental healthcare systems in the country and close the mental health treatment gap observed in our data by developing culturally-appropriate cost-effective accessible campus-based mental health interventions which are acceptable to students. One possibility for achieving this may be the use of e-interventions.

There are a number of limitations to this study, including the fact that we used cross-sectional data from a self-selected sample of students from two well-resourced universities. The participation rate was low and we relied on self-report data about mental healthcare utilisation. This low participation rate may have been a result of the length of the survey instrument, which took between 40 and 45 min to complete. Nonetheless, the limitations raise questions about the generalisability of the findings and highlight the importance of subsequent studies to verify these findings in large more representative samples of SA students.

## Conclusion

The data from this study points to a high unmet need for mental healthcare treatment of common mental disorders and suicidality among first-year university students in SA. These data point to sociodemographic inequalities in mental healthcare utilisation among SA university students which need to be addressed through: (1) research to understand inequalities in service utilisation; (2) targeted outreach programmes to promote treatment seeking among male, Black, and first-generation students with atypical sexual orientation; and (3) the implementation of services which are culturally-appropriate and acceptable to these sub-groups of students.

## Supplementary information

**Additional file 1: Table S1.** Interactions between the sociodemographic correlates of twelve-month treatments, among first-year university students in SA (n = 1402). **Table S2.** Multivariate analysis of sociodemographic predictors of twelve-month treatment seeking and 2 × 2 interactions (n = 1402). **Table S3.** Multivariate analysis of sociodemographic predictors of twelve-month treatment seeking including interaction terms (n = 1402). **Table S4.** Multivariate analysis of sociodemographic predictors of twelve-month treatment seeking including interaction terms (n = 1402). Detailed results of the analysis of sociodemographic and mental predictors associated with treatment seeking.

## Data Availability

Due to ethical restrictions, the data cannot be made publicly available. The datasets used and/or analysed during the current study are available from the corresponding author on reasonable request.

## Abbreviations

ADHD: attention deficit hyperactivity disorder
Army STARRS: Army Study to Assess Risk and Resilience in Service Members
AUD: alcohol use disorder
CMD: common mental disorder
DUD: drug use disorder
GAD: generalised anxiety disorder
MDD: major depressive disorder
SA: South Africa
SPSS: statistical package for social sciences
WHO: World Health Organisation
WMH-CIDI: Composite International Diagnostic Interview used in the World Mental Health Surveys
WMH-ICS: World Mental Health International College Student Initiative
US: United States of America
95% CI: 95% confidence interval
