# Supplementary material for: Inequality and mental healthcare utilisation among first-year university students in South Africa
Source: Int J Ment Health Syst. 2020 Jan 25;14:5. doi: 10.1186/s13033-020-0339-y (PMC6982378; doi:10.1186/s13033-020-0339-y)
Supplement: Supplementary file 1 — Additional file 1: Table S1. Interactions between the sociodemographic correlates of twelve-month treatments, among first-year university students in SA (n = 1402). Table S2. Multivariate analysis of sociodemographic predictors of twelve-month treatment seeking and 2 × 2 interactions (n = 1402). Table S3. Multivariate analysis of sociodemographic predictors of twelve-month treatment seeking including interaction terms (n = 1402). Table S4. Multivariate analysis of sociodemographic predictors of twelve-month treatment seeking including interaction terms (n = 1402). Detailed results of the analysis of sociodemographic and mental predictors associated with treatment seeking. [file 13033_2020_339_MOESM1_ESM.docx]

**Additional file 1**

**Table S1.** Interactions between the sociodemographic correlates of twelve-month treatments, among first-year university students in SA (n = 1402)

|  | Predictor distribution^a^ | 12-Month Treatment | |
| --- | --- | --- | --- |
|  | (95%CI) | aOR (95%CI) | |
| Gender (female) | 55.2% | **2.12*** | |
|  | (52.6-57.8) | **(1.38-3.26)** | |
| Race (black) | 41.4% | 0.69 | |
|  | (38.8-44.0) | (0.37-1.29) | |
| First generation students | 19.7% | 1.27 | |
|  | (17.7-21.9) | (0.47-3.41) | |
| Sexual orientation (atypical sexual orientation) | 22.2% | **2.37*** | |
|  | (20.1-24.5) | **(1.27-4.43)** | |
| Disability | 18.4% | **2.32*** | |
|  | (16.4-20.5) | **(1.16-4.63)** | |
| Gender X Race | 24.0% | 1.07 | |
|  | (21.8-26.3) | (0.55-2.12) | |
| Gender X First generation student | 10.3% | 1.41 | |
|  | (8.8-12.0) | (0.55-3.59) | |
| Gender X Sexual orientation | 11.7% | 0.91 | |
|  | (10.1-13.5) | (0.45-1.83) | |
| Gender X Disability | 12.8% | 0.62 | |
|  | (11.1-14.7) | (0.29-1.35) | |
| Race X First generation students | 14.6% | 0.56 | |
|  | (12.8-16.6) | (0.24-1.33) | |
| Race X Sexual orientation | 12.2% | 0.99 | |
|  | (10.5-14.0) | (0.49-1.98) | |
| Race X Disability | 7.6% | 0.78 | |
|  | (6.3-9.1) | (0.36-1.70) | |
| First generation students X Sexual orientation | 9.0% | **0.23*** | |
|  | (7.6-10.6) | **(0.09-0.59)** | |
| First generation students X Disability | 4.1% | 0.35 | |
|  | (3.1-5.3) | (0.11-1.11) | |
| Sexual orientation X Disability | 3.9% | 1.33 | |
|  | (2.9-5.1) | (0.58-3.09) | |
|  | | R2= 0.091 |  |
|  |  | X2 (15) = 80.28 |  |
|  |  | **p = 0.00*** |  |
| aOR = adjusted odds ratio | | |  |
| CI = confidence interval | | |  |
| ***p<0.05** | | |  |

**Table S2:** Multivariate analysis of sociodemographic predictors of twelve-month treatment seeking and 2X2 interactions (n=1402)

|  | Predictor distribution | 12-month treatment seeking |
| --- | --- | --- |
|  | (95%CI) | aOR (95% CI) |
| Gender (female) | 55.2% | **2.07*** |
|  | (52.6-57.8) | **(1.53-2.78)** |
| Race (black) | 41.4% | **0,62*** |
|  | (38.8-44.0) | **(0,46-0,85)** |
| First generation students | 19.7% | 0.94 |
|  | (17.7-21.9) | (0.56-1.56) |
| Sexual orientation (atypical sexual orientation) | 22.2% | **2.37*** |
|  | (20.1-24.5) | **(1.65-3.41)** |
| Disability | 18.4% | 1.40 |
|  | (16.4-20.5) | (0.99-1.96) |
| First generation students X sexual orientation | 9.0% | **0.23*** |
|  | (7.6-10.6) | **(0.09-0.55)** |
|  | | R^2^= 0.081 |
|  |  | X^2^ (6) = 71.54 |
|  |  | **p = 0.00*** |
| aOR = adjusted odds ratio | | |
| CI = confidence interval | | |
| ***p<0.05** | | |

**Table S3:** Multivariate analysis of sociodemographic predictors of twelve-month treatment seeking including interaction terms (n=1402)

|  | Predictor distribution | 12-month treatment seeking |
| --- | --- | --- |
|  | (95%CI) | aOR (95% CI) |
| Gender (female) | 55.2% | **2.07*** |
|  | (52.6-57.8) | **(1.53-2.78)** |
| Population group (black) | 41.4% | **0.62*** |
|  | (38.8-44.0) | **(0.46-0.85)** |
| Disability | 18.4% | 1.40 |
|  | (16.4-20.5) | (0.99-1.96) |
| Atypical sexual orientation versus typical sexual orientation among **second-generation students** | 16.4% | **2.37*** |
|  | (14.3-18.7) | **(1.65-3.41)** |
| First generation versus second-generation students with **typical sexual orientation** | 13.7% | 0.94 |
|  | (11.7-15.9) | (0.56-1.56) |
| First generation versus second-generation students with **atypical sexual orientation** | 40.6% | **0.21*** |
|  | (35.1-46.3) | **(0.10-0.44)** |
|  | | R^2^= 0.081 |
|  |  | X^2^(6) = 71.54 |
|  |  | **p = 0.00*** |
| aOR = adjusted odds ratio | | |
| CI = confidence interval | | |
| ***p<0.05** | | |

**Table S4:** Multivariate analysis of sociodemographic predictors of twelve-month treatment seeking including interaction terms (n=1402)

|  | Predictor distribution | 12-month treatment seeking |
| --- | --- | --- |
|  | (95%CI) | aOR (95% CI) |
| Gender (female) | 55.2% | **2.07*** |
|  | (52.6-57.8) | **(1.53-2.78)** |
| Population group (black) | 41.4% | **0.62*** |
|  | (38.8-44.0) | **(0.46-0.85)** |
| Disability | 18.4% | 1.40 |
|  | (16.4-20.5) | (0.99-1.96) |
| First-generation versus second-generation students with **typical sexual orientatio**n | 13.7% | 0.94 |
|  | (11.7-15.9) | (0.56-1.56) |
| Atypical sexual orientation versus typical sexual orientation among **first generation students** | 45.7% | 0.54 |
|  | (39.7-51.8) | (0.24-1.20) |
| Atypical sexual orientation versus typical sexual orientation among **second-generation students** | 16.4% | **2.37*** |
|  | (14.3-18.7) | **(1.65-3.41)** |
|  | | R^2^= 0.081 |
|  |  | X^2^(6) = 71.54 |
|  |  | **p = 0.00*** |
| aOR = adjusted odds ratio | | |
| CI = confidence interval | | |
| ***p<0.05** | | |
